# Modeling Root Zone Effects on Preferred Pathways for the Passive Transport of Ions and Water in Plant Roots

**DOI:** 10.3389/fpls.2016.00914

**Published:** 2016-06-23

**Authors:** Kylie J. Foster, Stanley J. Miklavcic

**Affiliations:** Phenomics and Bioinformatics Research Centre, School of Information Technology and Mathematical Sciences, University of South AustraliaMawson Lakes, SA, Australia

**Keywords:** Casparian strip, suberin lamellae, elongation zone, differentiation zone, transpiration, salt uptake

## Abstract

We extend a model of ion and water transport through a root to describe transport along and through a root exhibiting a complexity of differentiation zones. Attention is focused on convective and diffusive transport, both radially and longitudinally, through different root tissue types (radial differentiation) and root developmental zones (longitudinal differentiation). Model transport parameters are selected to mimic the relative abilities of the different tissues and developmental zones to transport water and ions. For each transport scenario in this extensive simulations study, we quantify the optimal 3D flow path taken by water and ions, in response to internal barriers such as the Casparian strip and suberin lamellae. We present and discuss both transient and steady state results of ion concentrations as well as ion and water fluxes. We find that the peak in passive uptake of ions and water occurs at the start of the differentiation zone. In addition, our results show that the level of transpiration has a significant impact on the distribution of ions within the root as well as the rate of ion and water uptake in the differentiation zone, while not impacting on transport in the elongation zone. From our model results we infer information about the active transport of ions in the different developmental zones. In particular, our results suggest that any uptake measured in the elongation zone under steady state conditions is likely to be due to active transport.

## 1. Introduction

Plant roots are responsible for the uptake of water and nutrients, as well as any potentially toxic ions, from the soil. This uptake varies along the longitudinal axis of roots, particularly across the different developmental zones, as a result of both changes in internal root anatomy (e.g., Zhou et al., 2011) and changes in the functional expression of transporters. There has been extensive investigation into the identification of ion transporters and their role in the uptake and transport of ions in plant roots (Barberon and Geldner, 2014). However, understanding how changes in root anatomy influence the transport of ions and water is also important for improving our understanding of root function. In this paper we utilize mathematical modeling to explore the effects of cell differentiation on the preferred pathways for the passive uptake of ions and water by plant roots. This model is an extension of previous efforts (Foster and Miklavcic, 2013, 2014), which have already highlighted the intrinsic coupling of ions in their transport through the root.

Plant roots can be divided longitudinally into anatomically distinct developmental zones, each of which have different abilities to take up and transport ions and water. For example, *Arabidopsis thaliana* primary roots consist of three distinct zones (Dolan et al., 1993): the meristematic zone, followed by the elongation zone (EZ) and then the differentiation zone (DZ). In the EZ the cells lengthen in preparation for differentiation, while in the DZ the cells become fully differentiated (Dolan et al., 1993). This process of cell differentiation can significantly affect the transport of ions and water. For example, in the primary development stage of the endodermis the Casparian strip (CS) develops in the cell wall of adjacent endodermal cells. The CS is a barrier that consists predominantly of lignin (Naseer et al., 2012) which blocks the passive uptake of ions (and possibly water) via the non-selective, apoplastic pathway (Geldner, 2013; Barberon and Geldner, 2014). In addition, the suberin lamellae (SL) forms in the secondary development stage of the endodermis. The SL forms between the plasma membrane and the primary cell wall of the endodermal cells. This barrier eventually covers the entire cell and inhibits the uptake of water and solutes via transporters across the plasma membrane of the affected endodermal cells (Geldner, 2013; Barberon and Geldner, 2014). On the other hand, the SL does not sever the plasmodesmatal connections between the cells. Hence, this disruption of the selective pathway forces solutes and water to enter the symplastic pathway in the outer regions of the root in order to enter the stele (Geldner, 2013).

Numerous experiments, conducted over many decades, have investigated the uptake of ions and water along the longitudinal axis of roots and how this uptake relates to root anatomy (e.g., Graham et al., 1974; Lüttge, 1983; Zhou et al., 2011). Similarly, experimental measurements of how transport properties (such as hydraulic conductivity) vary across different developmental zones have been carried out (e.g., Melchior and Steudle, 1993; Frensch et al., 1996). In addition, experiments have been conducted to identify the cell-specific distribution of ions across roots in order to gain information about transport mechanisms, including the importance of root structures such as the endodermal barriers as control points in the uptake process (e.g., Lauchli et al., 2008). An improved understanding of this uptake has important implications for better understanding root function, including the transport of nutrients and the response of plants to various stresses. Clearly, the spatial distribution of ion transporters has a significant influence on the uptake of ions along the length of the root as well as on the distribution of ions within the root. However, the focus of this work is on the effects of cell differentiation on uptake. Direct experimental testing of the roles of anatomically distinct root zones has proved difficult (Barberon and Geldner, 2014). Genetic advances, such as the recent identification of *sgn3*, an *Arabidopsis* mutant with a nonfunctional CS (Pfister et al., 2014), are important. To complement these experimental efforts mathematical modeling can provide an opportunity to examine in detail the spatial distribution of ion and water fluxes, as well as ion concentrations, and how these vary over time. Modeling also provides the ability to explore directly the effects of cell differentiation on ion and water transport. For example, the role of endodermal barriers can be examined by comparing model scenarios where the barriers are present to those where the barriers are absent (e.g., Foster and Miklavcic, 2014; Sakurai et al., 2015). Modeling also allows us to determine the relative contributions of different driving forces, such as diffusion and convection, to the overall transport process. Finally, it is relevant to consider how much of the observed ion uptake and concentration distributions can be explained by passive processes. This would be difficult or impossible to explore experimentally, whereas modeling can simulate scenarios in which uptake in a root occurs by passive processes without the interference of active transporters.

Previous simulations of solute uptake by plant roots have typically investigated radial, rather than axial, variations in root transport properties. For example, Claus et al. (2013) and Sakurai et al. (2015) developed models of the uptake of zinc and silicon, respectively, in the DZ. Their efforts focused on the radial distribution of these solutes (including the effects of apoplastic transport barriers) and as a result they did not consider any variations in transport properties along the longitudinal axis of the root. In contrast, Shimotohno et al. (2015) modeled the uptake and spatial distribution of boron in a plant root, including axial variation in the model root transport properties to simulate different root developmental zones. The emphasis of the simulations conducted by Shimotohno et al. (2015), however, was on the root tip and hence the effects of endodermal barriers and functional xylem were not simulated. All of the transport models described above included transport via membrane transporters. However, none of these models simulated transport in a plant root which had a zone of undifferentiated cells as well as a zone in which functional xylem and endodermal barriers were present.

Convection is likely to be an important driving force for ion transport, particularly in the DZ where functional xylem is present. However, convection is often not modeled in root ion transport simulations. An exception is the zinc model developed by Claus et al. (2013) which included convection in the symplast via a constant water flow rate. Claus et al. (2013) identified that this convection was an important factor affecting the radial zinc concentration pattern. However, this model did not incorporate all of the interactions between solutes and water. In particular, the osmotic effects of ion concentrations on water transport were not modeled.

In this study we extend our compartmental model of ion and water transport in a plant root (Foster and Miklavcic, 2013, 2014) to simulate different developmental zones along the longitudinal axis of the root. We include both the EZ and the DZ, incorporating the transition from non-functional to functional xylem and the development of the endodermal barriers. In contrast to the ion transport models discussed above, we focus on passive transport and include interactions between ion and water transport. As assumed previously (Foster and Miklavcic, 2013, 2014), we do not explicitly consider the apoplastic and symplastic pathways separately. Instead, the transport parameters, fluxes and concentrations are a composite representation of both pathways.

Using our model, we identify that the main steady state, passive, uptake of ions and water occurs at the start of the DZ. In contrast, the passive uptake in the EZ at steady state is negligible due to the insignificant level of convection in this region. From this we conclude that any significant uptake in the EZ under steady state conditions is likely to be driven by active transport. In addition, we show that the level of transpiration has a significant effect on the pattern of ion distribution (both radially and axially) in the DZ and on the rate of ion and water uptake into the aerial parts of the plant. This has important implications for both modeling and experimental efforts.

## 2. Model of water and ion transport

### 2.1. Model root structure

**Figure 1:**
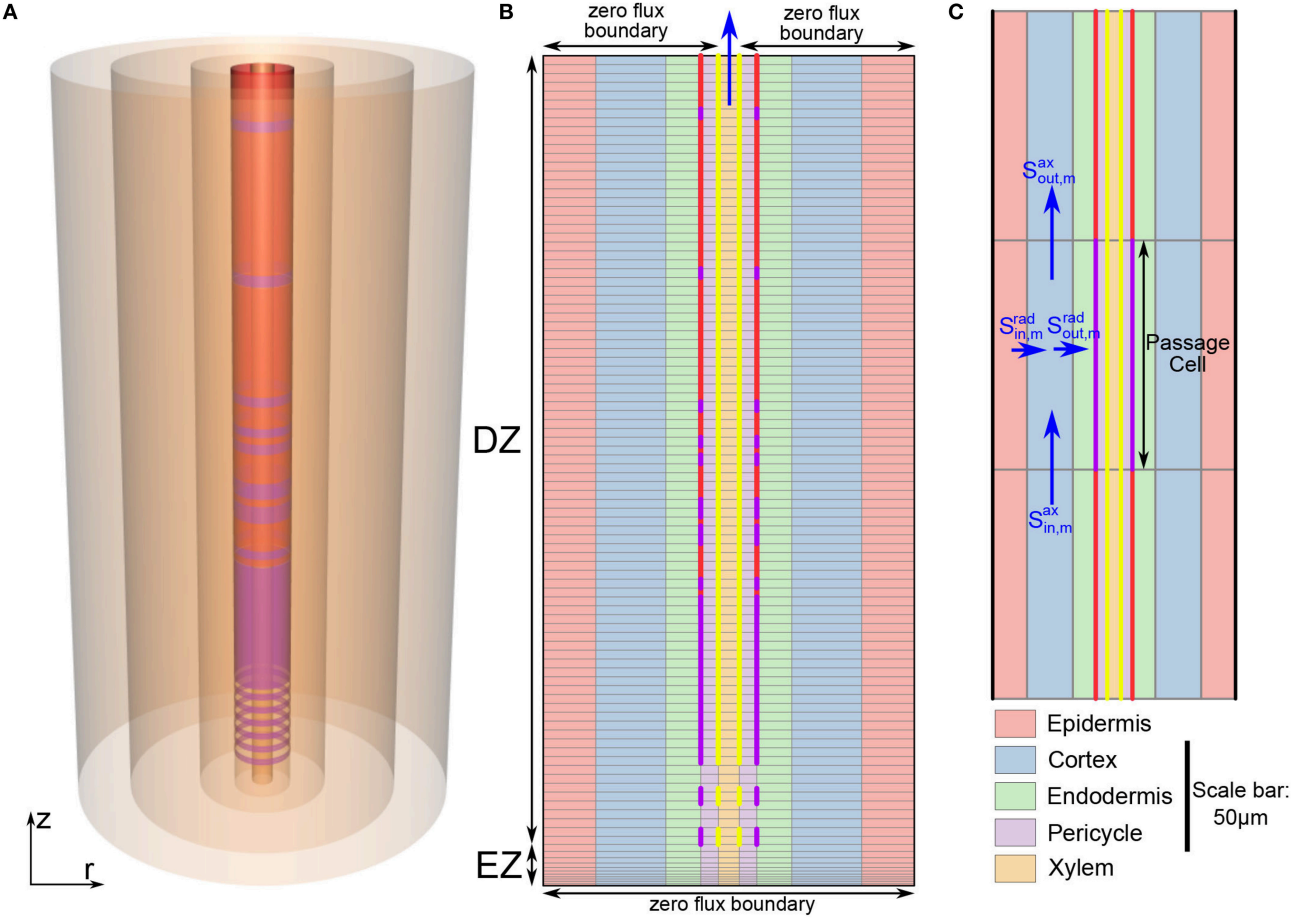
**(A)** Schematic of the model root showing the radial discretization used to represent the different root tissue types (from outermost region to innermost region: the epidermis, cortex, endodermis, pericycle, and xylem). The purple and red cylinders show the location of the CS and SL, respectively. **(B)** Schematic longitudinal cross-section of the model root. The light gray lines show the radial and axial discretization. The different tissue types are highlighted by different colors, while the extent of different developmental zones (EZ and DZ) are indicated by arrows. Some of the model boundary conditions are also indicated. For visual clarity, the *z* and *r* axes are scaled differently. **(C)** Expanded view of a small section of the root cross-section in **(B)** showing the location of a passage cell. The *z* and *r* axes are here drawn to the same scale (refer to the scale bar) highlighting the actual relative axial to radial proportions. The blue arrows indicate ion fluxes. In **(B,C)**, vertical purple lines indicate the location of the CS, vertical red lines represent SL and yellow lines represent functional xylem. Purple dashed lines indicate incomplete CS while yellow dashed lines represent the transition from non-functional to functional xylem. The root dimensions are given in Table 1. The coordinate system (*r, z*) is as shown in **(A)**. Gravity force is in the direction of decreasing *z*.

In this compartmental model we simulate the passive uptake and transport of multiple ions and water through a plant root. The model root consists of a single, flat ended, right cylinder (Figure 1A). The anatomy of the root is modeled on the structure of *Arabidopsis* primary roots. The model includes radial variation in transport properties to represent the different tissue types, as well as longitudinal variation in transport parameters to represent the different developmental zones (the EZ and DZ). Due to the compartmental nature of the model, the root is discretized radially into consecutive annular cylinders which represent the different tissue types present in *Arabidopsis* roots: the epidermis, cortex, endodermis, pericycle and xylem (see Figure 1). In addition, the model root is further discretized along the longitudinal axis with the height of each compartment reflecting the height of an individual cell. Hence, the height of each compartment varies across the EZ (see Figure 1B).

For simplicity we assume axial symmetry. Therefore, the features of the model root are independent of the angle around the central axis, instead they depend only on the radial and axial coordinates. As a result, any modeled disruption of the endodermal barriers (e.g., due to the presence of passage cells) is represented as a disruption of the barrier for the full circumference of the endodermis at the relevant location along the length of the root (see Figure 1A).

As an extension of our previous work (Foster and Miklavcic, 2013, 2014) both axial and radial flow of water and solutes is modeled in all of the root tissues. This allows us to investigate the effects of changes in root anatomical structure that occur along the length of the root. To capture the interactions between water and ion transport, we model their uptake as a coupled system. Radial and axial flows of water occur due to both osmotic and hydraulic pressure gradients, while both radial and axial flows of ions are driven by local concentration differences, electric field effects and convection. This is a compartmental model in which the root is divided into model compartments (see Figures 1B,C), with the hydrostatic pressure and concentration of each ion assumed to be uniform throughout each model compartment. The discretization of the root into compartments is described in further detail in the Supplementary Material. As this is a composite model water and ion fluxes are not explicitly separated into apoplastic and symplastic contributions. Instead, the transport parameters, fluxes and concentrations are composites of both pathways. This model does not explicitly include transport via active transporters. To our advantage, it can be used to examine how much of the observed behavior of ions in plant roots can be explained by passive processes.

The focus of this report is on the transport of solutes up into the plant rather than transport of any solutes (such as sucrose) down into the root. Hence, we assume that the transpiration stream occurring in the xylem is the dominant function of the vascular bundle.

### 2.2. Forces driving ion and water transport

In this section we outline the methods used to model ion and water transport. The detailed model equations are provided in the Supplementary Material.

We assume that the root consists of rigid and completely water filled compartments. The radial and axial flow of water in tissue regions where cell membranes are present is driven by both hydraulic and osmotic pressure gradients. Hence, the flow rate of water in these regions is modeled using non-equilibrium thermodynamics (Katchalsky and Curran, 1965). We use the van't Hoff relation to calculate the osmotic pressure in these regions (Katchalsky and Curran, 1965). In contrast, axial transport in the mature xylem is not interrupted by cell membranes and hence, the axial flow of water in the xylem is driven by hydraulic pressure gradients only. This water flow is modeled as linearly proportional to the hydraulic pressure gradient, using Darcy's Law.

The radial and axial transport of ions in the model plant root is governed by a chemical potential contribution (arising from concentration differences), an electric field contribution (due to electric potential differences) and by convection. This is modeled using the extended Nernst-Planck equation (van der Horst et al., 1995). The electric potential is determined by solving Poisson's equation as described previously (Foster and Miklavcic, 2013, 2014). The transport of ions and water are interdependent due to both the contribution of water transport to the convection of the ions as well as the contribution of the ions to the osmotic pressure driving water transport.

### 2.3. Model parameters

Table 1 summarizes the parameter values used in the model simulations. The boundary conditions are described in Section 2.4.

**Table 1:** **Summary of parameters used and their source**.

**Parameter**	**Value**
Root hydraulic conductivity, *L*_*p*_	5.5 × 10^−8^ ms^−1^MPa^−1^ (Unless otherwise stated)
Solute permeability, *k_m_*	3 × 10^−9^ ms^−1^ (Unless otherwise stated)
Reflection coefficient, σ_*m*_	0.5 (Unless otherwise stated)
Axial xylem water permeability, *k^ax^*	5 × 10^−13^ m^2^
Dynamic viscosity, μ	1.002 × 10^−3^ Pa s
Water density, ρ	1.0 × 10^3^ kg m^−3^
Relative permittivity, ϵ_*r*_	80
Thickness of epidermis	15 μm
Thickness of cortex	20 μm
Thickness of endodermis	10 μm
Thickness of pericycle	5 μm
Radius of xylem	3 μm
Concentration of ions in soil	100 mM

Unless otherwise stated the transport parameters in the radial and axial directions are assumed to be equal. Root hydraulic conductivity was obtained from Javot et al. (2003), Ranathunge and Schreiber (2011), and Sutka et al. (2011). Solute permeabilities were obtained from Ranathunge and Schreiber (2011). Reflection coefficients were obtained from Boursiac et al. (2005) and Ranathunge and Schreiber (2011). Axial water permeability in the xylem was calculated using Poiseuille's Equation. The thicknesses of the root sections were obtained from Dolan et al. (1993), Scheres et al. (1995), Mattsson et al. (1999), Casimiro et al. (2003), and Javot et al. (2003).

The values of several model transport parameters were used to model the anatomical changes that occur across the different root tissues and root developmental zones. For example, the increased resistance to transport due to the CS and subsequently the SL were represented by changing the value of transport parameters at the endodermis-pericycle interface in the DZ, without changing the values adopted for the other tissue layers. In particular, the reduced diffusion due to these barriers was modeled by decreasing the radial diffusive permeability of each ion (*k*^*rad*^) by an order of magnitude for each barrier. Simultaneously, the reduced water transport and convection due to these barriers was modeled by decreasing the radial water permeability ($L_{p}^{rad}$) by an order of magnitude and increasing the radial reflection coefficient of each ion (σ^*rad*^) by 0.2 units for each barrier.

The functional CS and xylem were assumed to begin at the 12th cell from the start of the EZ (Naseer et al., 2012). To represent the observed gradual, patchy appearance of the CS in the early stages of endodermal cell differentiation (Roppolo et al., 2011) *k*^*rad*^, $L_{p}^{rad}$ and σ^*rad*^ were changed gradually across several axial model compartments from values equal to the surrounding tissues to values representing the fully formed, continuous CS. This region of developing CS is represented by dashed purple lines in Figure 1B. Similarly, the development of the xylem from non-functional to functional xylem in the transition from the EZ to the DZ was modeled by changing the axial water permeability ($L_{p}^{ax}$) and the axial reflection coefficient of each ion (σ^*ax*^) across several axial model compartments (dashed yellow lines in Figure 1B).

In the early stages of secondary endodermal cell differentiation, the SL is patchy. As the root matures the SL becomes a more continuous barrier, with the presence of only an occasional passage cell (Geldner, 2013). To model this observed behavior we assumed that the probability of the existence of a passage cell (i.e., a cell unaffected by SL) at a given location decreased along the length of the root. To achieve this, we used a scalar random generator to assign each endodermal cell as either a passage cell (with a probability of $\text{exp}\left(-\frac{j}{30}\right)$) or a cell affected by SL (with a probability of $1-\text{exp}\left(-\frac{j}{30}\right)$). Here *j* refers to the number of cells from the start of the EZ. The possibility of the SL first appearing was assumed to occur starting from the 38th cell from the start of the EZ (Naseer et al., 2012). This process provided a realistic distribution of passage cells (see Figure 1B).

### 2.4. Computational details

The discretization of our model root is discussed in detail in the Supplementary Material. However, one major difference between our current model and our previous root model (Foster and Miklavcic, 2013, 2014) is that the axial discretization of the root includes a region of varying compartment height to model the EZ (see Figure 1B).

The nonlinear, coupled system of ordinary differential equations, resulting from the model setup described in Section 2.2 and the Supplementary Material, was solved numerically in MATLAB using the ode15s package. Poisson's equation was solved numerically using finite difference methods to determine ψ as described in Foster and Miklavcic (2014). The hydraulic pressure in each compartment was determined at each time step by solving a linear system of equations as described in Foster and Miklavcic (2014).

The boundary conditions in the soil consist of a linear hydrostatic hydraulic pressure gradient and a fixed bulk concentration of four monovalent ions (two monovalent cation-anion pairs). The root is assumed to be vertical (see Figure 1), with the top of the root aligned with the surface of the external medium. Hence, as a result of the assumption of a hydrostatic pressure gradient outside the root, the pressure in the external medium at the top of the root is atmospheric, with this external pressure increased by ρ × *g*×*L* at the bottom of the root (here, ρ is the density of water, *g* is the acceleration due to gravity and *L* is the length of the root). These ideal boundary conditions assume that the root is surrounded by an infinite reservoir of both mobile ions and water and hence can be thought to be representative of the external conditions in, say, hydroponics experiments. In contrast, under actual field conditions there is usually a limited supply of water and solutes in the soil. Deviation from these ideal conditions may occur in practice for various reasons, such as non-uniform soil conditions, water depletion in the soil due to environmental factors (global effects) and uptake into the root (local effects). These non-uniform boundary effects could be included in future simulations by including the simultaneous modeling of water and ion transport in the soil. However, the focus of our work currently is on the response within the root and hence we have chosen to adopt the simplest of external conditions. Our model establishes a basic understanding of the root transport response under optimal external conditions. There is zero flux of water and ions across the bottom boundary of the root (as shown in Figure 1B).

The boundary conditions across the top of the root represent the connectivity of the root to the aerial sections of the plant. It is clear that the axial flow of water and ions up through the xylem into the stem should be significant and hence a zero flux boundary condition across the top of the root would not be appropriate. However, it is less clear how much flow occurs axially out of the other tissue types. Hence, we carried out model simulations for a range of boundary conditions at the top boundary of the model root. These included: axial flux of water and ions out of only the xylem with constant concentrations and hydrostatic pressure at the xylem boundary; axial flux out of only the xylem with a constant hydrostatic pressure and no concentration gradients across the xylem boundary; identical boundary conditions across the top of the root for all tissue types with constant concentrations and pressure at the boundary; and identical boundary conditions across the top of the root for all tissue types with a constant pressure and no concentration gradients across the boundary. We explored a wide range of values for the fixed hydrostatic pressure as well as the ion concentrations at the boundary. The pressure at the top boundary of the root (*P*_*b*_) significantly affected the simulation results. This is unsurprising since *P*_*b*_ simulates the level of transpiration, which is one of the key driving forces in our model. However, the remaining changes to the boundary conditions that were investigated led to only negligible changes in the simulation results, with only the fluxes and ion concentrations in the top three cell layers of the root affected. For the simulations shown in Section 3 we assume there is zero flux axially out of the outer four tissue regions at the top boundary of the root and there is a constant pressure and no concentration gradients across the top of the xylem (see Figure 1B). In Section 3 we explore the effects of different *P*_*b*_ values, which represent the effects of different transpiration conditions.

For the majority of the simulations the root is initially empty of ions. However, as found in our previous work (Foster and Miklavcic, 2013) the steady state model results are independent of the initial conditions. In all instances the root is completely filled with water.

## 3. Results

### The endodermal barriers significantly inhibit the passive influx of ions and water into the DZ

**Figure 2:**
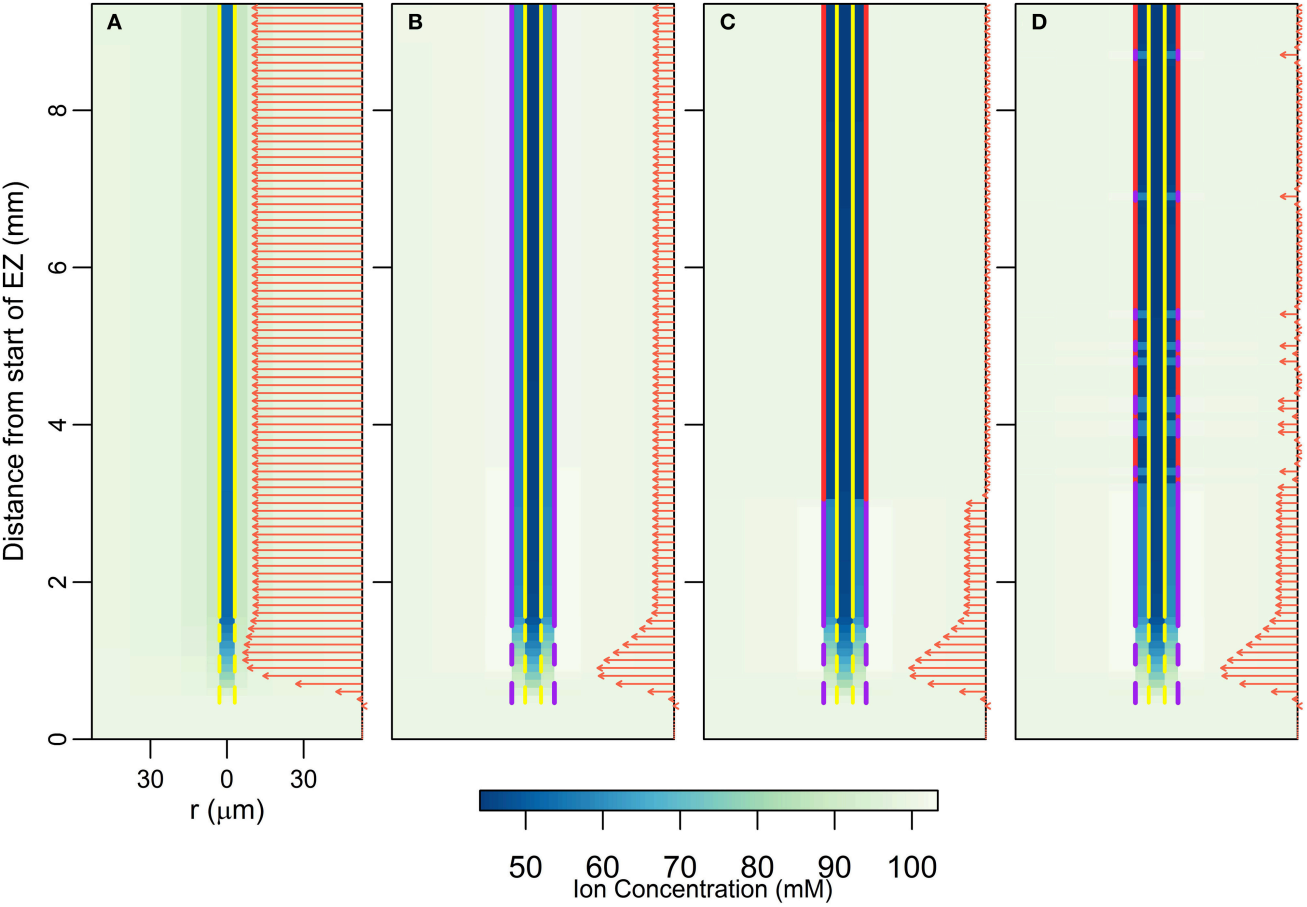
**Plots of steady state ion concentrations (colormaps) and ion fluxes (arrows) for four different root structures: (A) no endodermal barriers present, (B) CS present, (C) CS and SL present, (D) CS and SL with passage cells present**. Yellow vertical lines show the presence of functional xylem with dashed lines near the root tip indicating the gradual increase in conductivity as the xylem develops. Purple vertical lines represent the presence of the CS; dashed lines near the root tip indicate the gradual appearance of the barrier. Red vertical lines show the location of the SL, with gaps identifying the location of passage cells. The arrows show the magnitude of net radial solute flux across the root surface (in mol s^−1^), with the same scale used across all four subfigures. These arrows are also indicative of the corresponding water flow rates. All simulations assume 100 mM of 2 monovalent cations and 2 monovalent anions in the soil (e.g., 100 mM NaCl and 100 mM KNO_3_). All ions are assumed to have identical transport properties and the transport parameters used are provided in Table 1. Refer to Section 2.3 for an explanation of the simulation of the endodermal barriers and xylem development. These simulations were carried out using *P*_*b*_ = −0.5 MPa.

Figure 2 shows the steady state ion concentration (colormap) and ion flux (arrows) results for different endodermal barrier scenarios. In Figure 2A the root is unprotected by endodermal barriers. In Figure 2B, the CS is present which blocks the apoplastic pathway at the endodermis. In Figure 2C, both the CS and a solid, uninterrupted SL barrier are present. Hence, both the apoplastic and selective transport pathways are affected at the endodermis. Finally, Figure 2D represents the most realistic scenario in which both the CS and the SL are present with a gradual introduction of the SL and passage cells occurring along the length of the root. At the passage cell locations the SL is absent but the CS is still present (Peterson and Enstone, 1996).

In all of the scenarios, there is no significant uptake (i.e., net radial flux across the root surface) of solutes or water at steady state in the EZ (see arrows in Figure 2). As there is no functional xylem in the EZ, there is no convection of ions further up into the root. The influence of the conductive xylem extends only a few cells into the EZ, and hence this region is hydraulically isolated from the rest of the root. Therefore, diffusion is the main mechanism of ion transport in this region. As a result, at steady state the concentration of ions in the EZ for all four scenarios is equal to the bulk concentration in the soil.

In contrast, the introduction of the endodermal barriers has a strong effect on the amount and location of ion and water uptake in the DZ (see arrows in Figure 2). When there are no endodermal barriers present, the main uptake of water and ions occurs throughout the region where the xylem is, or is close to, fully functional (Figure 2A). However, when the endodermal barriers are present, the main uptake occurs closer to the root tip where the CS is not yet fully developed (giving an average net radial ion flux of 15.19 nmol s^−1^m^−2^ across the root surface in this region at steady state for the scenario shown in Figure 2D), despite the fact that in this region the xylem is *not* fully functional (Figures 2B–D). The SL substantially blocks water and solute uptake with an average ion flux of 0.58 nmol s^−1^m^−2^ across the root surface in the region where the SL is fully developed (excluding passage cell locations) compared to 6.73 nmol s^−1^m^−2^ in the region where the CS is fully developed (Figures 2C,D). However, at the location of passage cells there is increased uptake, with an average ion flux across the root surface of 5.71 nmol s^−1^m^−2^ at their location (Figure 2D). This enhanced uptake supports the idea that passage cells function as key entry points into the stele (Peterson and Enstone, 1996; Andersen et al., 2015). The role of passage cells is explored further in the following sections.

In terms of ion concentrations, the main effects of the introduction of each barrier include an increase in the concentration gradient across the barrier, as well as a decrease in the concentration gradient across the tissues inside the barrier (i.e., the pericycle-xylem interface). Both of these changes result from relatively large reductions in the concentrations of ions in the pericycle, with smaller changes in concentrations in the xylem and endodermis also occurring. The introduction of the first barrier (the CS) causes the greatest change, with the introduction of a further barrier (the SL) having a less significant impact.

The results shown in Figure 2 were simulated using a pressure boundary of −0.5 MPa at the top of the root (see Section 2.4 for further details about the boundary conditions). However, the discussion in this section is valid at lower transpiration conditions. The effects of different transpiration conditions are discussed in the following sections.

### The endodermal barriers reduce the rate of ion and water uptake into the shoot, with greater reductions at higher transpiration rates

**Figure 3:**
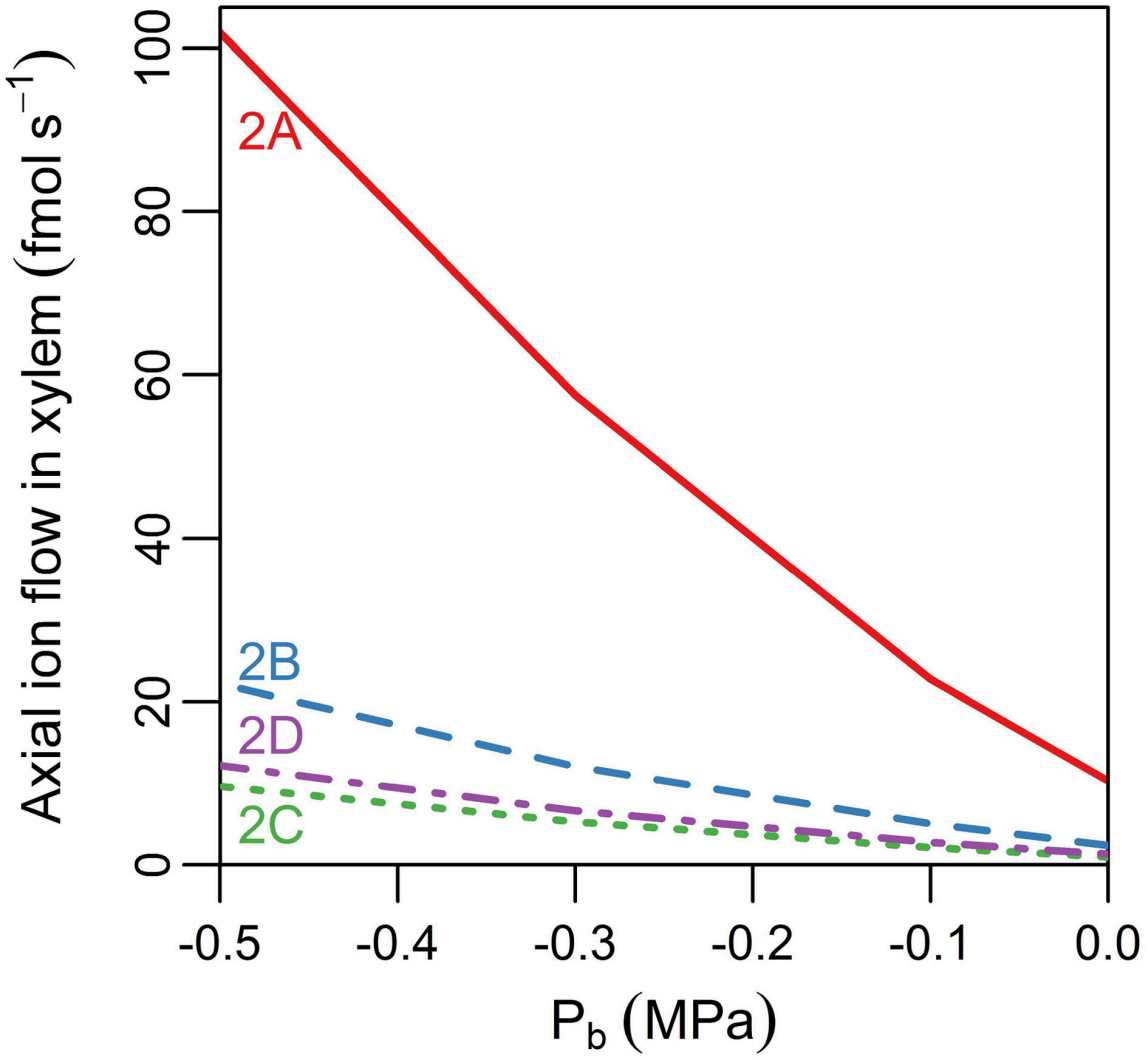
**Line plot showing the steady state axial flux of ions out of the top of the root xylem as a function of pressure at the top of the root, ***P***_***b***_ (representing different rates of transpiration), and of the barriers present in the endodermis (different line types)**. The scenarios considered are: no endodermal barriers (solid red line); CS only (dashed blue line); both CS and SL present with no passage cells (green dotted line); and both CS and SL with passage cells present (purple dot-dashed line). The labels indicate the corresponding scenarios in Figure 2. The axial fluxes into the xylem compartment three cells down from the top of the root were used to minimize the influence of the flux boundary conditions chosen at the top of the root. The solute fluxes shown are also indicative of the corresponding water flow rates. All simulations assume 100 mM of 2 monovalent cations and 2 monovalent anions in the soil. All ions are assumed to have identical transport properties and the transport parameters used in the simulations are provided in Table 1.

Considering the relatively small changes in ion concentrations in the xylem, with the introduction of the endodermal barriers (Figure 2), it may at first glance appear that these barriers have minimal impact on the transport occurring within the xylem. However, other changes in root function occur in response to the introduction of each barrier. In particular, there are substantial decreases in both axial ion and water flow rates in the xylem (Figure 3). Importantly, it is this rate of axial transport out of the top of the root that determines the amount of ions and water entering the stem from the root.

The introduction of each endodermal barrier leads to reduced transport of both ions and water from the root into the aerial parts of the plant (Figure 3). The effect of each barrier becomes more significant as the transpiration rate increases (represented by a more negative *P*_*b*_). These results show the compromise inherent in the function of the endodermal barriers: the entry of harmful ions into the xylem must be minimized while still maintaining the uptake of beneficial ions and water. The model simulations show a small increase in the axial flow rates of ions and water at the top of the root due to the presence of passage cells (see Figure 3, purple dot-dashed line vs. green dotted line), with a higher number of passage cells resulting in a larger increase (results not shown). These results support the idea that one function of passage cells is to enhance the uptake of water and at least some ions (Peterson and Enstone, 1996).

### Delayed development of CS relative to functional xylem significantly enhances passive ion and water uptake

**Figure 4:**
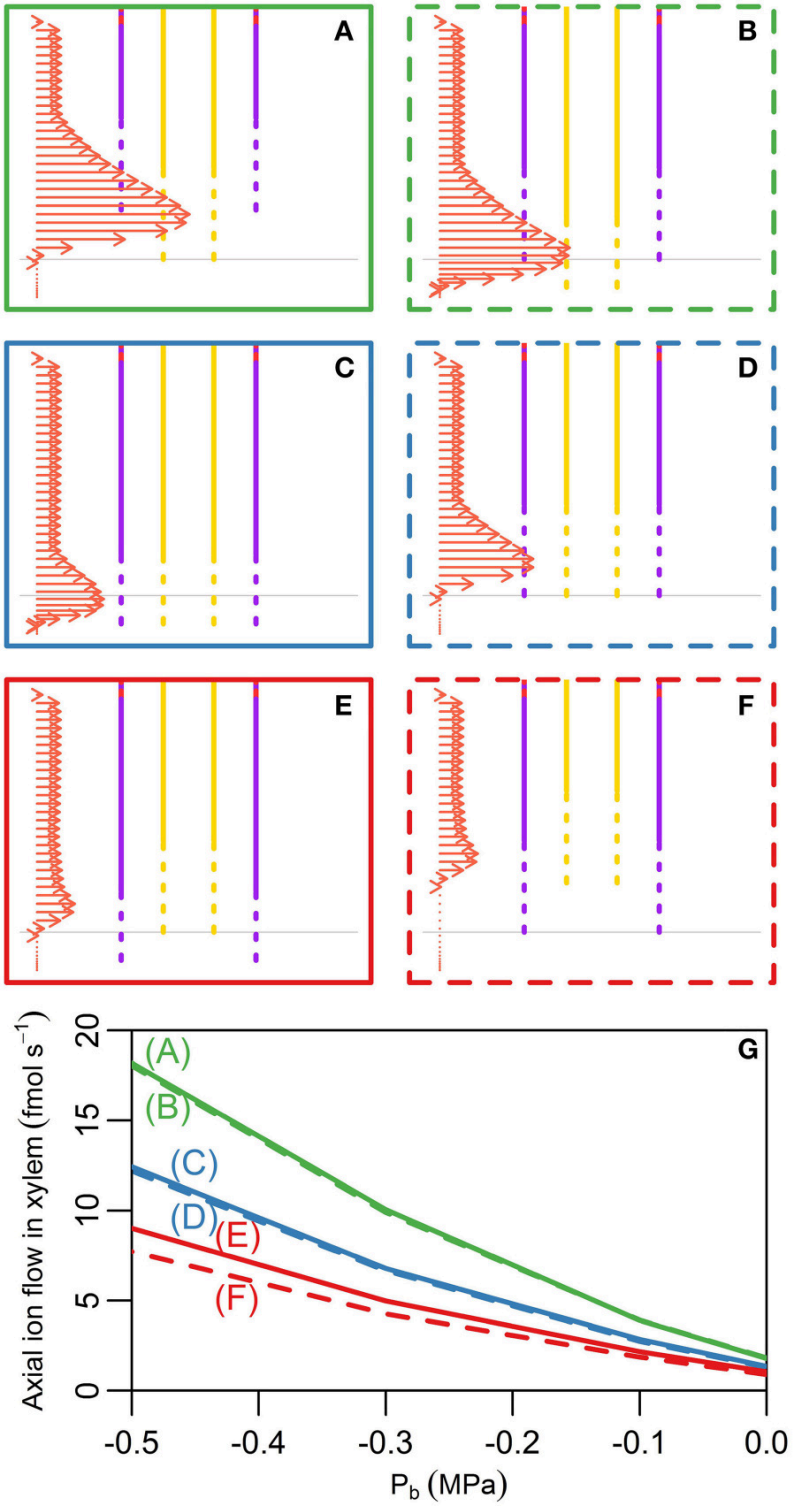
**(A–F)** Plots showing the lower section of the model root, indicating the different initial positions of development of the CS and functional xylem that were explored. Purple (yellow) vertical lines indicate the presence of the CS (functional xylem). Dotted lines represent the gradual development of the CS and xylem, while solid lines represent fully functioning CS and xylem. Horizontal gray lines show the location of the start of the CS and xylem used in the model simulations shown in all other figures. The arrows show the net radial solute flux across the surface of the root at steady state for each scenario, these flows are indicative of the solute fluxes into the xylem. The same scale is used for the arrows across all six subfigures. Although these arrows show solute fluxes, they are also indicative of the corresponding water flow rates. The arrows shown represent the model results for *P*_*b*_ = −0.3 MPa. **(G)** Line plots showing the steady state axial solute fluxes out of the top of the model xylem as a function of the pressure boundary condition (representing the transpiration rate) and the relative positions of the initial development of the CS and xylem. The axial fluxes into the xylem compartment three cells down from the top of the root were used to minimize the influence of the type of flux boundary conditions chosen at the top of the root. The line colors and styles in **(G)** match the colors and styles of the boxes around the corresponding scenario in **(A–F)**. All simulations assume 100 mM of 2 monovalent cations and 2 monovalent anions in the soil. All ions are assumed to have identical transport properties and the transport parameters used in the simulations are provided in Table 1.

In *Arabidopsis* primary roots, the xylem has been observed to appear in conjunction with the CS (Alassimone et al., 2010). We have thus far assumed that the development of the CS and functional xylem is coincident along the length of the root (Figure 2). However, exposure to various stresses (e.g., salinity) can influence the development of the endodermal barriers (Enstone et al., 2002) as well as the xylem (Cruz et al., 1992; Reinhardt and Rost, 1995), altering their position relative to the root tip and to each other. In Figure 4 we explore the physiological effects of shifting the first points of development of the xylem and CS, both together and relative to each other. Figures 4A–F show the different locations considered, with vertical lines showing the positions of the CS (purple lines) and functional xylem (yellow lines). The arrows indicate the ion fluxes across the root surface, but are also representative of the water flow rates. Although the results in Figures 4A–F assume *P*_*b*_ = −0.3 MPa, the qualitative behavior is very similar for other transpiration conditions; the peak in uptake, however, becomes less pronounced as the transpiration rate decreases. Figure 4G shows the rate of ion transport out of the top of the root xylem for the scenarios shown in Figures 4A–F for a range of transpiration conditions. The different positions shown in Figures 4A–F have only a minimal effect on the concentration of the ions in the root xylem at the top of the model root (results not shown).

A key result is that it is the *relative* location of the first points of development of the xylem and the CS that has the largest effect on the axial transport of water and ions up out of the root (Figure 4G). If any length of xylem is unprotected by the endodermal barrier, due to either delayed development of the CS (Figure 4A) or early maturation of the xylem (Figure 4B), there is increased transport of water and ions radially into the root and hence out of the xylem at the top of the root (green lines in Figure 4G). In contrast, if the CS develops before the xylem (Figures 4E,F) this transport is reduced (red lines in Figure 4G).

These results suggest that experiments examining the development of endodermal barriers could also quantify the development of the xylem and the relative positions of the two. In particular, examining the relative development of the CS and xylem and how this correlates with applied stresses could provide useful insights into the stress response of plants. For example, salt stress has been observed to result in the endodermal barriers developing closer to the root tip due to a reduction in the root growth rate as well as accelerated maturation of the barriers (Enstone et al., 2002). The observed reduced root growth rate would presumably also lead to the xylem developing closer to the root tip. However, the accelerated maturation rate of the barriers could lead to the CS developing closer to the root tip than the xylem (similar to the scenario shown in Figure 4E). This could be a favorable adaptation to salt stress as it would result in reduced passive uptake of ions (Figure 4G), although it would also lead to reduced water uptake. Similarly, when comparing the development of endodermal barriers across species or varieties in order to explain differences in their transport abilities it would seem important to consider the relative development of the xylem and CS.

Altered development of the SL can be correlated with altered development in the CS (Enstone et al., 2002). Hence, we also explored scenarios representing this correlation. We found similar behavior to that shown in Figure 4, with the additional feature that any reduction in the total length of the SL resulted in increased axial flow of ions in the xylem (results not shown).

In all of the scenarios considered, the peak uptake of water and solutes into the root occurs in the region where the xylem is present and either the CS is not present or is still developing. This agrees with the experimentally observed pattern of rapid uptake of water in the young region of the root (Graham et al., 1974; Clarkson and Robards, 1975). In contrast, the experimentally observed uptake of ions is more variable (Lüttge, 1983). This variability is unsurprising due to the variation in the types and locations of ion specific transporters. However, for some ions, peak uptake has been observed in this young region of the root; an example of this is calcium (Robards et al., 1973; White, 2001). It is worth noting that the modeled peak in ion uptake is not significantly affected even if the ions have differing diffusive permeabilities (results not shown). Our results show that the location of the peak in uptake is less obvious if the CS extends beyond the xylem (compare Figures 4E,F to Figures 4C,D) or if the transpiration level is low (see the next section). Hence, differences in the anatomy of plant roots as well as experimental conditions may affect the extent and prominence of the peak in uptake.

The model results in Figures 4A–F show the locations of peak uptake. However, they do not specify which region of the root contributes the most to the total uptake of ions and water. Due to the longer length of the mature region of the root compared to the short length of the high uptake region, the mature region may contribute more to the overall uptake of ions and water. The presence of lateral roots also contributes to the uptake in the more mature regions of the root.

### Low vs. high transpiration leads to significantly different spatial distributions of ions in the DZ, but not in the EZ

**Figure 5:**
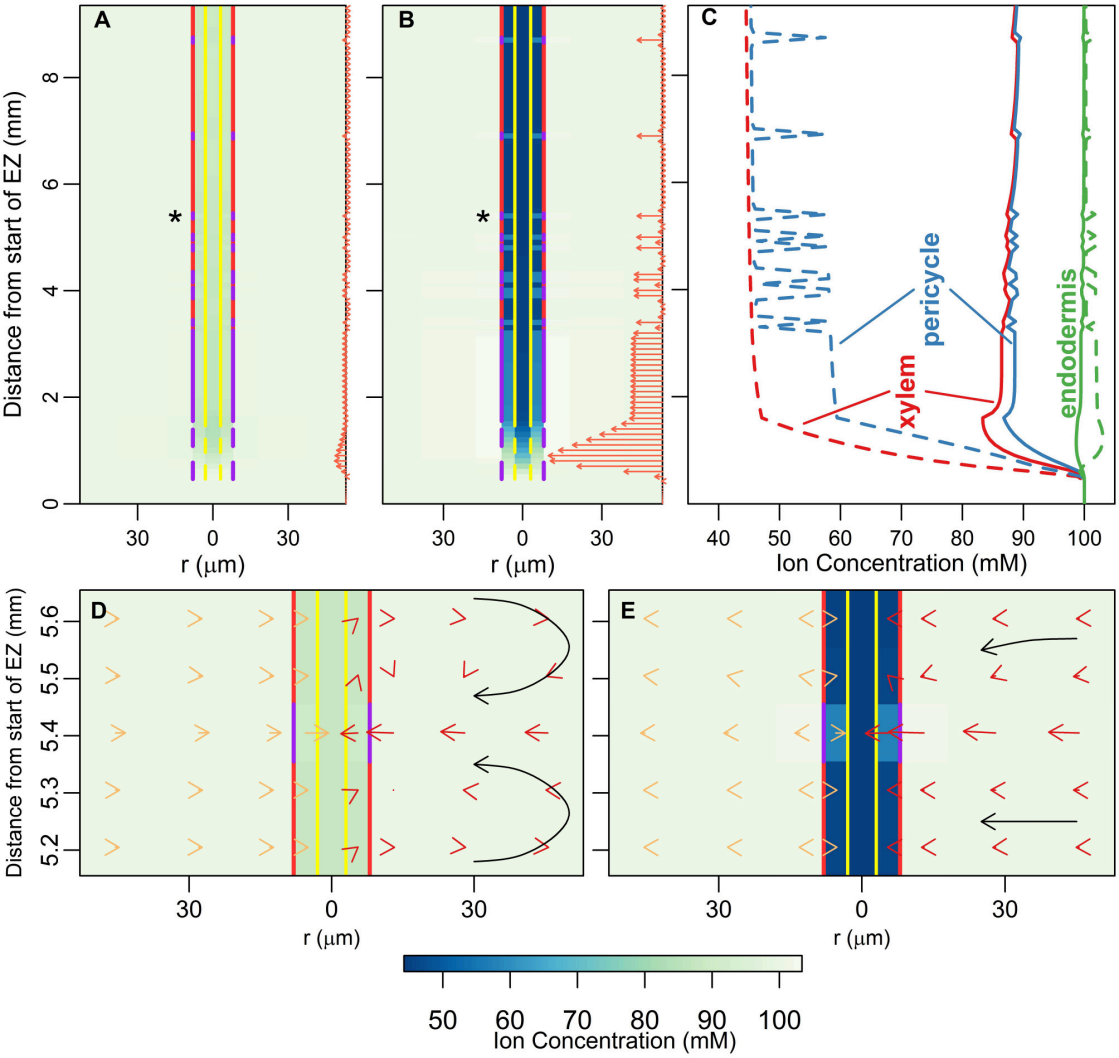
**(A,B)** Plots of steady state ion concentrations (colormaps) and ion fluxes (arrows) for two different transpiration conditions: *P*_*b*_ = 0 MPa and *P*_*b*_ = −0.5 MPa respectively. The arrows are also indicative of water flow rates. The same scale is used for the arrows in **(A,B)**. **(C)** Line plots of ion concentrations in **(A)** (solid lines) and **(B)** (dotted lines). The line colors refer to tissue regions: xylem (red lines), pericycle (blue lines) and endodermis (green lines). **(D,E)** show zoomed-in sections of results in **(A,B)**, respectively, at the location of the passage cell indicated by asterisks. Yellow arrows on the left show the diffusive component of the total ion flow, red arrows on the right show the convective component (adding the two together gives the total flow). The arrows are shown in the center of their corresponding model compartment. The arrows in **(D,E)** are drawn to different scales for clarity, an equal length arrow in **(E)** represents a 10 times larger flux than in **(D)**. The black arrows highlight the convective flow path and are not drawn to scale. All simulations assume 100 mM of 2 monovalent cations and 2 monovalent anions in the soil. All ions are assumed to have identical transport properties and the transport parameters used in the simulations are provided in Table 1.

Figures 3, 4G initiated a study of the effect of transpiration on the rate of ion and water uptake. In this section we explore in greater detail the influence of transpiration on the distribution of ions within the root (Figure 5). The effect of varying the transpiration level is particularly felt in the DZ, with the level of transpiration influencing both the axial and radial distribution of ions.

The qualitative behavior of the axial distribution of ions in the DZ varies depending on the level of transpiration. Under high transpiration there is a steady decline in concentration in the xylem along the length of the root in the DZ (Figure 5B and red dotted line in Figure 5C), whereas under low transpiration conditions the ion concentrations in the xylem increase along the length of the root in the DZ (Figure 5A and solid red line in Figure 5C). This dichotomy arises from the competing effects of diffusion and convection. High transpiration results in ions being convected away faster than can be replaced by diffusion, while under low transpiration the low level of convection cannot offset the influx of ions by diffusion into the xylem from the surrounding tissues.

In terms of the radial distribution of ions in the DZ, the concentration of ions in the xylem and pericycle is lower at higher levels of transpiration (Figure 5B and dotted lines in Figure 5C compared to Figure 5A and solid lines in Figure 5C) due to greater convection up and out of the root. In addition, at higher levels of transpiration there is a build up of ions in the endodermis at locations where only the CS is present (including at passage cell locations), while this build up is not evident at lower transpiration levels (green dotted lines compared to solid lines in Figure 5C). Figures 5D,E highlight how the interactions between diffusion and convection generate these contrasting results. In particular, where only the CS is present, a high level of convection (red arrows in Figure 5E) is required to offset the process of diffusion, which counteracts the formation of any concentration gradients (see yellow arrows in Figure 5E). In contrast, at low levels of transpiration, the inward convection of ions is small or even reversed due to osmotic pressure gradients (red arrows in Figure 5D) and hence there is no build up of ions outside the endodermal barriers. Overall these results point to the importance of considering the effect of the level of transpiration when experimentally investigating the function of endodermal barriers, particularly when using the presence of a build up of ions outside the CS as an experimental measure of how well it is functioning as a barrier (e.g., Lauchli et al., 2008).

In contrast to the results in the DZ, the steady state concentrations in the EZ are identical and equal to the ion concentrations in the soil for both transpiration scenarios (see Figures 5A–C). Since there is only negligible convection in the EZ, transpiration conditions do not influence the uptake of ions in this hydraulically isolated region of the root. Similarly, for both transpiration scenarios the concentration of ions in the xylem and pericycle decreases through the transition zone from the EZ to the DZ due to the development of a functional xylem. However, this decrease is more pronounced under higher transpiration conditions (see Figures 5A–C).

The effect of passage cells on the distribution of ions extends across several tissue regions (see Figure 5C). There are higher levels of both diffusion and convection across the endodermis in the vicinity of passage cells compared to the surrounding cells which are affected by the SL (Figures 5D,E). Where there are passage cells, ions build up in the pericycle as both diffusion and convection draw ions in from the surrounding outer tissues (see the yellow diffusive and red convective arrows in Figures 5D,E). Again, these results confirm the function of passage cells as key entry points into the stele (Peterson and Enstone, 1996).

The arrows in Figures 5A,B show that the pattern of ion *uptake* is not qualitatively affected by the level of transpiration. However, the magnitude of ion uptake is strongly dependent on the rate of transpiration.

The significant differences in the distribution of ions for the different transpiration conditions are supported by experimental observations. For example, Møller et al. (2009) found that the measured radial distribution of Na^+^ and K^+^ differed substantially between plants grown in conditions where there was little or no transpiration and those plants grown in transpiring conditions. In addition, our predicted increase in the magnitude of water uptake along the length of the root for higher transpiration conditions (Figures 5A,B) has also been observed experimentally (e.g., Sanderson, 1983).

### Measurements of transient vs. steady state ion fluxes reflect different aspects of root function

**Figure 6:**
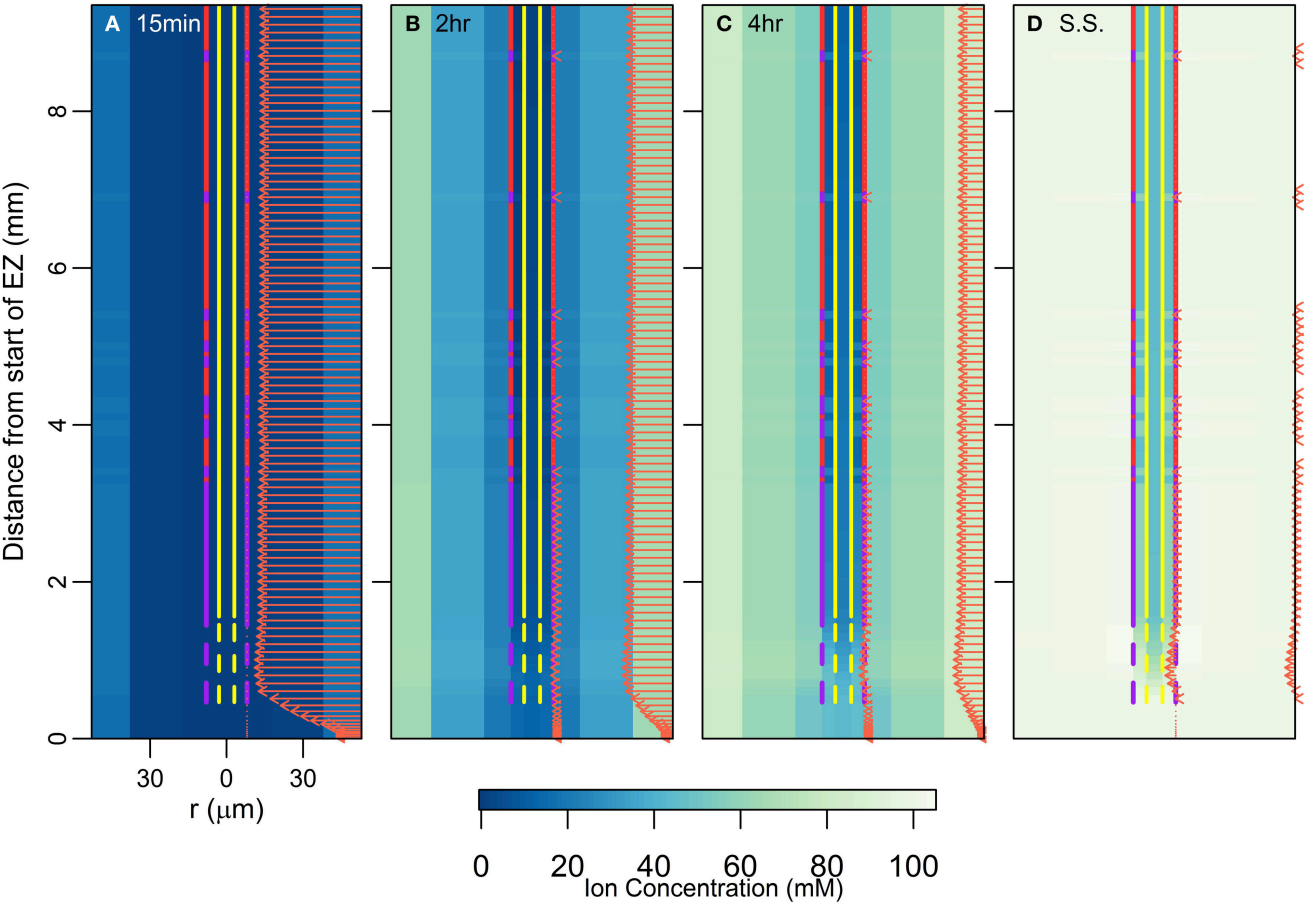
**Plots of transient ion concentrations (colormap) and ion fluxes (arrows) for the root structure shown in Figure 2D**. For clarity only the net radial solute fluxes across the root surface (into the epidermis) and across the endodermis-pericycle interface are shown. The initial condition for this simulation was the steady state result for a simulation carried out with 100 mM of 1 monovalent cation and 1 monovalent anion in the soil (e.g., KNO_3_). The simulation was then carried out assuming 100 mM of 2 monovalent cations and 2 monovalent anions in the soil (e.g., the existing KNO_3_ with NaCl added). The results shown are for the added ions. The results show the changes over time after the ions were added: **(A)** 15 min after, **(B)** 2 h after, **(C)** 4 h after, **(D)** at steady state (approximately 1 day after). These simulations were carried out using P_*b*_ = −0.5 MPa. All ions are assumed to have identical transport properties and the transport parameters used in the simulations are provided in Table 1.

The design of experiments to measure the fluxes of ions and/or water across root surfaces (e.g., Zhou et al., 2011) should incorporate an appropriate time frame to match the purpose of the experiment, i.e., are transient or steady state conditions appropriate? Transient ion fluxes demonstrate which regions along the length of the root fill up most rapidly, while steady state fluxes indicate the regions through which the main uptake occurs over time. This is highlighted in Figure 6, which shows how the ion concentrations (colormap) and the radial solute fluxes (arrows) change over time. For clarity only the net radial solute fluxes into the epidermis and pericycle are shown in Figure 6, rather than the fluxes into all of the root tissues. As in previous figures, the arrows in Figure 6 also represent the water flow rates, which are in direct proportion to the ion fluxes. The results indicate that the solute fluxes at the surface are not representative of those in the inner regions of the root until the system is at or near steady state (Figures 6A–C). However, when the system is at steady state (Figure 6D), solute fluxes across the root surface are representative of where the main uptake of ions into the inner regions occurs. This is provided there are no large vertical fluxes in the intervening regions. Further complications in flux measurements would arise if the ions being measured are converted to other forms before reaching the stele (e.g., the reduction of nitrate to nitrite).

## 4. Discussion

Our work focuses on the passive uptake of ions and water, in particular, exploring the interaction between the processes of diffusion and convection. The results therefore show the ion uptake patterns and concentration distributions that can be explained by passive processes and their interaction with cell differentiation. However, using these results one can also infer information about the active transport of ions. In particular, our results describe the pattern of ion and water uptake that occurs when only passive processes operate (arrows in Figure 2D) and highlight that qualitatively this pattern is relatively unaffected by either differences in the level of transpiration (arrows in Figures 5A,B) or differences in cell development (Figures 4A–F), although the magnitudes can be substantially affected. Hence, an ion uptake pattern that qualitatively differs substantially from Figure 2D would indicate the operation of an active transport mechanism. For example, any significant uptake of ions in the EZ at steady state is likely to be driven by active processes as there is only minimal passive uptake in this region (see arrows in Figures 2D, 6D). Similarly, any significant uptake of water in this region is likely to be driven by osmotic pressure gradients developed by the active transport of ions.

It is no surprise that the level of transpiration has a very significant effect on the rate of ion and water uptake (Figures 3, 4G and arrows in Figure 5), as well as on the spatial distribution of ions radially and axially inside the DZ of the root (Figure 5). Indeed, the level of impact that transpiration has on the spatial distribution of ions is of a similar scale to the level of impact of the presence of the endodermal barriers (compare Figures 2, 5). Our results support the experimental findings of Møller et al. (2009) that different transpiration conditions lead to differences in the radial distribution of ions across plant roots. In addition, our results highlight the fact that some transport behavior becomes more distinct as the level of transpiration increases, e.g., the influence of the endodermal barriers on ion uptake (Figure 3), as well as the positions of peak uptake (Figures 5A,B). Hence, our findings suggest that the results of ion and water measurements conducted on non-transpiring plants or excised roots (with no suction applied to cut end) are likely to differ substantially from measurements conducted on intact, transpiring plants. In particular, the effects of convective processes will be more obvious under higher transpiring conditions, whereas under low or non-transpiring conditions the effects of active processes are likely to be more obvious, although under all growth conditions the transport will also be affected by diffusion. These differences need to be considered when attempting to compare the results of experiments conducted under different transpiration conditions (see e.g., Møller and Tester, 2007).

The effects of the transpiration level also have ramifications for other modeling efforts. Firstly, our more detailed simulations of water transport concur with previous modeling findings that the level of convection is a significant factor in determining the radial distribution of ions (Claus et al., 2013). Convection is clearly an essential mechanism to include in models of ion transport in roots in which functional xylem is present. Secondly, the distinctly different patterns of ion distribution resulting from differing transpiration levels (Figures 5A–C) suggest the importance of investigating the interactions between convective and membrane transport processes in any study of the effects of membrane transporters on the pattern of solute distribution (e.g., Claus et al., 2013; Sakurai et al., 2015). Thirdly, convection does not seem to be significant in regions of root where the xylem is not conductive, such as in the EZ. In our case, the zone of influence of the xylem extended only a few cells in the direction of the root tip from the initial conductive cells. These findings support earlier assumptions that it is unnecessary to include convection when modeling ion transport in regions where the xylem is not functional (e.g., Shimotohno et al., 2015). Hence, while it is not necessary for convection to be incorporated into models of ion transport in the meristematic zone and the EZ, it may be very important for convection to be considered in the more mature regions of the root.

It has been suggested that passage cells function as low resistance pathways for the uptake of water and at least some ions (Peterson and Enstone, 1996). Our findings (Figures 2D, 3, 5) give this position some support. This role has been suggested due to the consistent positioning of passage cells adjacent to protoxylem poles (Peterson and Enstone, 1996). However, there is currently very limited direct experimental evidence identifying the function of passage cells (Andersen et al., 2015). To investigate the role of passage cells further it would be necessary to differentiate between the apoplastic and symplastic pathways.

Our efforts to model transport within a single root do not include contributions from lateral roots to the uptake of ions and water. Not only do lateral roots increase the root surface area available for uptake, their formation also interrupts the CS (Vermeer et al., 2014), potentially allowing leakage of ions and water across the endodermis. Hence, the influx of ions and water at the location of lateral root formation would likely be similar to the modeled influx found at the location of passage cells (e.g., Figure 2D).

In this paper we have explored passive transport processes. However, to investigate the uptake and transport of specific ions (such as K^+^ or Na^+^) it is necessary to incorporate models of active transport, such as the model we have developed recently (Foster and Miklavcic, 2015). Such a challenge requires the explicit modeling of symplastic and apoplastic pathways, which would allow us to investigate more specific aspects of cell differentiation that cannot be included in the current model. For example, one of the suspected functions of the CS is to prevent backflow of ions out of the stele under low transpiration conditions (Enstone et al., 2002). An investigation of this process would require active transport mechanisms to be included since it is thought that active transport is responsible for the build up of ions in the xylem under low or non-transpiring conditions (Enstone et al., 2002). In addition, a more complex model would allow a clearer separation of the function of the CS (which blocks the apoplastic pathways) from the function of the SL (which is thought to block uptake into the symplastic pathway), including a further exploration of the role of passage cells.

## 5. Conclusions

In this paper we have presented the results of simulations of the transport of ions and water in a plant root via passive transport processes. The model root incorporates both different tissue types and different developmental zones. As in our previous works (Foster and Miklavcic, 2013, 2014), the model includes the self-consistent interaction between the transport of ions and water. We have used the model to simulate a wide range of transport scenarios and found that, in all instances where the endodermal barriers were present, the peak uptake of water and ions occurred at the start of the DZ. In addition, we found that there was no substantial uptake in the EZ at steady state due to the lack of functional xylem in this region. From this we infer that any observed uptake of ions (or water) in this developmental zone under steady state conditions is likely to be due to active transport processes. The results also highlight that the level of transpiration has a significant impact on water and ion transport in the DZ. This should be taken into consideration both when conducting experiments and developing models to examine transport in this developmental zone.

We have used our model of passive transport to infer information about active transport processes. However, more detailed modeling is required to examine the transport of specific ions. For example, combining a single cell model of active and passive transport of ions as well as water, which we have developed previously (Foster and Miklavcic, 2015), with the root model presented here would allow more detailed exploration of ion transport (specifically Na^+^, K^+^, and Cl^−^) across the different developmental zones. For example, it would allow an examination of how each of the endodermal barriers influence the different transport pathways. This is the subject of ongoing efforts. Nevertheless, the passive processes of diffusion and convection modeled in this paper are fundamental to ion uptake. Hence, the results discussed in this paper establish a baseline of transport phenomenon on which active transport mechanisms are imposed and with which active transport mechanisms interact.

## Author contributions

The authors were equal contributors to the design of simulations, analysis of results and drafting of the paper. KF performed the simulations.

## Funding

This project is supported by an Australian Postgraduate Award and a Grains Industry Research Scholarship from the Grains Research and Development Corporation for KF.

### Conflict of interest statement

The authors declare that the research was conducted in the absence of any commercial or financial relationships that could be construed as a potential conflict of interest. The reviewer ID and handling Editor declared their shared affiliation, and the handling Editor states that the process nevertheless met the standards of a fair and objective review.
