# Supplementary material for: Modeling Root Zone Effects on Preferred Pathways for the Passive Transport of Ions and Water in Plant Roots
Source: Front Plant Sci. 2016 Jun 23;7:914. doi: 10.3389/fpls.2016.00914 (PMC4917552; doi:10.3389/fpls.2016.00914)
Supplement: Supplementary file 1 [file DataSheet1.pdf]

Supplementary Material: Modeling Root Zone Effects on Preferred Pathways for the Passive Transport of Ions and Water in Plant Roots

Kylie J. Foster and Stanley J. Miklavcic *
*Correspondence:
Stanley Miklavcic
stan.miklavcic@unisa.edu.au

1 GOVERNING EQUATIONS

For rigid and completely water-filled compartments with constant fluid density, mass conservation of solution requires the condition

$$Q_{in}^{ax} - Q_{out}^{ax} + Q_{in}^{rad} - Q_{out}^{rad} = 0, \tag{S1}$$

where Q_{in}^{ax} (Q_{out}^{ax}) is the *axial* volume flow rate of solution into (out of) position (r, z), and Q_{in}^{rad} (Q_{out}^{rad}) is the *radial* volume flow rate into (out of) position (r, z).

The concentration of solute species m at position (r, z) is given by a conservation equation:

$$\frac{dC_m}{dt} = \frac{1}{V}\left[S_{in,m}^{ax} - S_{out,m}^{ax} + S_{in,m}^{rad} - S_{out,m}^{rad}\right], \tag{S2}$$

where V is the solution volume, C_m is the concentration of species m, S_{in}^{ax} (S_{out}^{ax}) is the *axial* flux of solute m into (out of) position (r, z) and S_{in}^{rad} (S_{out}^{rad}) is the *radial* flux of solute m into (out of) position (r, z).

The flow rate of water in a plant root is driven by both hydraulic and osmotic pressure gradients. Hence, the radial flow rate of water is given by (Katchalsky and Curran, 1965):

$$Q_{in}^{rad} = L_p^{rad} A^{rad}\left[\Delta p^{rad} - R_g T \sum_{m=1}^{N} \sigma_m^{rad} \Delta C_m^{rad}\right], \tag{S3}$$

and similarly, the axial flow rate of water in all tissues *except* the region in which the xylem is conductive is given by,

$$Q_{in}^{ax} = L_p^{ax} A^{ax}\left[\Delta p^{ax} - \rho g \Delta z - R_g T \sum_{m=1}^{N} \sigma_m^{ax} \Delta C_m^{ax}\right], \tag{S4}$$

where L_p^{rad} (L_p^{ax}) is the position-dependent, radial (axial) water permeability, A^{rad} (A^{ax}) is the element of surface area through which the radial (axial) water flow is occurring, Δp is the hydraulic pressure gradient across the region of interest, ρ is the fluid density, g is the acceleration due to gravity directed toward

decreasing z, Δz is a discrete height increment (see Section 2.4 for further details), R_g is the Universal Gas Constant (8.314 J mol^{-1}K^{-1}), T is the (constant) temperature, σ_m^{rad} (σ_m^{ax}) is the position-dependent radial (axial) reflection coefficient of solute ion $m = 1, \ldots, N$. Here, N is the total number of ions in the system; ΔC_m is the concentration difference of ion type m across the region of interest. In Eqs. (S3) and (S4) we have assumed that solute concentrations are sufficiently low that use of the van't Hoff relation for osmotic pressure, $\Delta\Pi^m = R_g T \Delta C_m$, is valid (Katchalsky and Curran, 1965). The appropriateness of this assumption is discussed in Foster and Miklavcic (2014).

In contrast to the other root tissue regions, axial transport in the functional xylem is not interrupted by cell membranes. Hence, the axial flow of water in the xylem is driven by hydraulic pressure gradients only, with no osmotic pressure gradients present. This water flow can be modeled as linearly proportional to the hydraulic pressure gradient, using Darcy's Law,

$$Q_{in}^{ax} = \frac{k^{ax} A^{ax}}{\mu} \frac{\Delta p^{ax} - \rho g \Delta z}{\Delta z}, \text{ in functional xylem} \quad \text{(S5)}$$

where k^{ax} is a position-dependent, axial water permeability, and μ is the (constant) dynamic viscosity of the fluid.

The transport of ions in the model plant root is governed by a chemical potential contribution (arising from concentration differences), an electric field contribution (due to an electric potential difference) and by convection. Hence, the radial flux of ions ($S_{in,m}^{rad}$) and the axial flux of ions in all tissues except the functional xylem ($S_{in,m}^{ax}$) are given by,

$$S_{in,m}^{rad/ax} = k_m^{rad/ax} A^{rad/ax} \times \left[\Delta C_m^{rad/ax} + \frac{Z_m C_m F}{R_g T} \Delta \psi^{rad/ax} \right] + \left(1 - \sigma_m^{rad/ax} \right) C_m Q_{in}^{rad/ax}. \quad \text{(S6)}$$

where, k_m^{rad} (k_m^{ax}) is the radial (axial) diffusive permeability of ion m, Z_m is the valence of ion m, F is Faraday's constant (96 485 C mol^{-1}), and $\Delta\psi$ is the electric potential gradient across the region of interest.

Due to the absence of membranes, the axial flux of solutes in the functional xylem is given by,

$$S_{in,m}^{ax} = k_m^{ax} A^{ax} \left[\Delta C_m^{ax} + \frac{Z_m C_m F}{R_g T} \Delta \psi^{ax} \right] + C_m Q_{in}^{ax}, \text{ in functional xylem} \quad \text{(S7)}$$

where the convection term is not scaled by a solute reflection coefficient.

The value of ψ in Eqs. (S6) - (S7) is determined by solving Poisson's equation as described previously (Foster and Miklavcic, 2013, 2014).

In order to implement the equations outlined above, the r and z root volume space was discretized into compartments identified by discrete positions (r_α, z_j), where $\alpha = 1, \ldots, 5$ and $j = 1, \ldots, 100$.

REFERENCES

Foster, K., Miklavcic, S. J., 2013. Mathematical modelling of the uptake and transport of salt in plant roots. Journal of Theoretical Biology 336, 132–143.

Foster, K. J., Miklavcic, S. J., 2014. On the competitive uptake and transport of ions through differentiated root tissues. Journal of Theoretical Biology 340, 1–10.

Katchalsky, A., Curran, P. F., 1965. Nonequilibrium Thermodynamics in Biophysics. Harvard University Press, Cambridge.
